# Hyperalgesic Effect Evoked by il-16 and its Participation in Inflammatory Hypernociception in Mice

**DOI:** 10.1007/s11481-024-10145-7

**Published:** 2024-08-17

**Authors:** Sara González-Rodríguez, Christian Sordo-Bahamonde, Alejandro Álvarez-Artime, Ana Baamonde, Luis Menéndez

**Affiliations:** 1 Laboratorio de Farmacología, Facultad de Medicina, Instituto Universitario de Oncología del Principado de Asturias (IUOPA), Universidad de Oviedo, C/ Julián Clavería 6, 33006 Oviedo, Asturias, Spain; 2 Departamento de Biología Funcional, Instituto Universitario de Oncología del Principado de Asturias (IUOPA), Universidad de Oviedo, C/ Julián Clavería 6, 33006 Inmunología Oviedo, Asturias, Spain; 3 Instituto de Investigación Sanitaria del Principado de Asturias (ISPA), Oviedo, Spain

**Keywords:** IL-16, Neutrophils, Pain, Mice, Inflammation

## Abstract

The systemic administration of interleukin-16 (IL-16, 3–30 ng/kg) induced thermal hyperalgesia in mice, that was prevented by the acute injection of an anti-CD4 antibody (1 µg/kg), the depletion of circulating white blood cells by cyclophosphamide or the specific reduction of circulating CD4^+^ cells provoked by a high dose of an anti-CD4 antibody (30 µg/mouse, 24 h before). IL-16-induced hyperalgesia was locally inhibited after intraplantar (i.pl.) administration of the non-selective cyclooxygenase (COX) inhibitor diclofenac, the COX-1 inhibitor SC-560, the COX-2 inhibitor celecoxib, the TRPV1 antagonist capsazepine or the TRPA1 antagonist HC030031, thus demonstrating that prostaglandins and TRP channels are involved in this effect. The i.pl. administration of low doses of IL-16 (0.1–1 ng) evoked local hyperalgesia suggesting the possibility that IL-16 could participate in hypernociception associated to local tissue injury. Accordingly, IL-16 concentration measured by ELISA was increased in paws acutely inflamed with carrageenan or chronically inflamed with complete Freund´s adjuvant (CFA). This augmentation was reduced after white cell depletion with cyclophosphamide or neutrophil depletion with an anti-Ly6G antibody. Immunofluorescence and flow cytometry experiments showed that the increased concentration of IL-16 levels found in acutely inflamed paws is mainly related to the infiltration of IL-16^+^ neutrophils, although a reduced number of IL-16^+^ lymphocytes was also detected in paws inflamed with CFA. Supporting the functional role of IL-16 in inflammatory hypernociception, the administration of an anti-IL-16 antibody dose-dependently reduced carrageenan- and CFA-induced thermal hyperalgesia and mechanical allodynia. The interest of IL-16 as a target to counteract inflammatory pain is suggested.

**Graphical Abstract:**

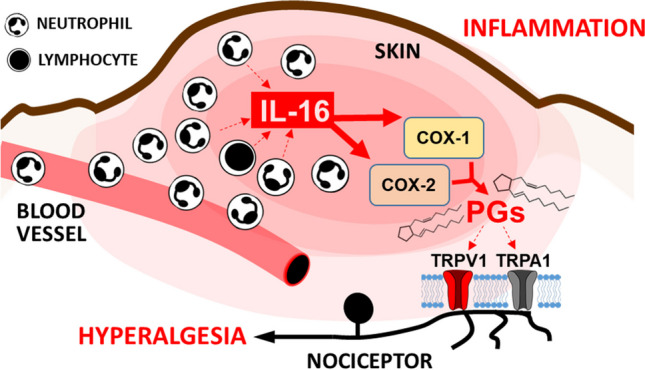

## Introduction

Besides their outstanding role in the immune response, different cytokines exert important effects in the modulation of nociception (Cook et al. 2018). Although most of them, as tumour necrosis factor (TNF)-α, interleukins IL-1ẞ or IL-6 (Cook et al. 2018) are associated to hyperalgesic responses, cytokines do not always behave as nociceptive amplifiers and, in fact, some interleukins (IL), as IL-4 or IL-10 (van Helvoort et al. 2022), promote analgesic responses and particular chemokines can even induce opposite effects depending on the dose, as occurs with CCL5 (González-Rodríguez et al. 2017a) or CCL4 (Aguirre et al. 2020). CCL4 is a notorious example of dual effects since the systemic administration of extremely low doses (pg/kg) induces analgesic effects (García-Domínguez et al. 2019) whereas remarkable hyperalgesic responses appear when doses are increased up to the range of ng/kg (Aguirre et al. 2020). White blood cells, particularly lymphocytes, and the release of different molecules able to produce inhibition or sensitization of nociceptors are involved in the establishment of the effects evoked by CCL4. As such, met-enkephalin seems to be the main molecule responsible for the analgesic action induced by low doses of IL-16 (García-Domínguez et al. 2019), whereas several hyperalgesic chemokines, as CCL2, CXCL1 or CXCL13 or the interleukin IL-1α, participate in the induction of hyperalgesia following the administration of higher doses of this chemokine (Aguirre et al. 2020). Although the hyperalgesic properties of these mediators had already been described, this study unmasked the participation in CCL4-induced hyperalgesia of IL-16, a cytokine for which its possible hypernociceptive function remained so far unknown. In fact, IL-16 seems an essential mediator in the hypernociceptive response evoked by CCL4 being its neutralization with an anti-IL-16 antibody enough to transform CCL4-induced hyperalgesia in opioid analgesia (Aguirre et al. 2020).

IL-16 is produced by different immune cells such as CD8^+^ and CD4^+^ T-lymphocytes, but also by other types of cells, such as neutrophils, monocytes, dendritic cells, mast cells, fibroblasts, microglia, endothelial cells, keratinocytes or neurons (Mathy et al. 2000; Skundric et al. 2015; Gillis et al. 2020; Roth et al. 2015). Due to its key role in the modulation of CD4^+^ T-lymphocytes (Center and Cruikshank 1982), it was initially named Lymphocyte Chemoattractant Factor and, in fact, its main molecular target is the CD4 (cluster of differentiation 4) receptor (Liu et al. 1999). By acting on this receptor, IL-16 can stimulate the migration and proliferation of CD4^+^ T-lymphocytes or other immune cells expressing it, such as a subset of CD4^+^ monocytes (Mathy et al. 2000), eosinophils (Rand et al. 1991), or dendritic cells (Kaser et al. 1999). In addition, IL-16 binding to CD4 can also modulate T cell receptor (TCR)/CD3-mediated responses, leading to anergy, a situation in which lymphocyte activation is suppressed (Theodore et al. 1996). Thus, even if IL-16 is more frequently considered a proinflammatory mediator, some authors have considered it as an anti-inflammatory molecule (Klimiuk et al. 1999), probably reflecting these diverse responses.

Although elevated levels of IL-16 have been found in very heterogeneous clinical settings, the study of the involvement of IL-16 in pathophysiological processes is rather incomplete. Thus, high concentrations of IL-16 have been detected in the blood of infants who developed cow's milk protein allergy (Mao et al. 2022), atopic dermatitis (Zheng et al. 2016), systemic sclerosis (Kawabata et al. 2020), chronic lymphocytic leukemia (Wu et al. 2023) and in the urine of patients with lupus nephritis (Häyry et al. 2022). Related to the central nervous system, IL-16 is expressed in immune cells or resident microglia and astrocytes during multiple sclerosis (Skundric et al. 2015; Hridi et al. 2021). More related to the context of our study that deals with inflammation, the role of IL-16 in rheumatoid arthritis is an issue widely considered. High levels of IL-16 have been found either in blood (Theodore et al. 1996; Blaschke et al. 2001) or synovial fluid (Franz et al. 1998; Kageyama et al. 2000; Blaschke et al. 2001) from arthritic patients and, in accordance, the overexpression of this cytokine has been demonstrated in peripheral blood mononuclear cells (ElAtta et al. 2019), neutrophils (De la Rosa et al. 2020) and synovial cells (Franz et al. 1998; Cho et al. 2008). These data suggest that IL-16 may act as an important stimulus for the recruitment of immune cells, specially CD4^+^ lymphocytes (Franz et al. 1998), although some attempts to correlate IL-16 expression with clinical severity have not offered solid conclusions (Kageyama et al. 2000; Blaschke et al. 2001). In any case, the properties of IL-16 could suggest that, besides its possible participation in the pathophysiology of this disease, its presence in arthritic joints could also contribute to nociceptive sensitization through the stimulation of the expression of other pro-inflammatory cytokines (Mathy et al. 2000) or even the direct stimulation of nociceptors (Kanngiesser et al. 2012).

Considering the scarce knowledge related to the hyperalgesic effect evoked by IL-16 and the interest of its possible participation in rheumatoid arthritis, we designed the present set of experiments to enhance our understanding relative to the pronociceptive actions evoked by IL-16 by elucidating whether it depends on the activation of CD4 receptor in immune cells and by exploring the possible role played by cyclooxygenase–derived mediators and TRP channels. Furthermore, we tried to determine whether IL-16 could be functionally relevant in the pathophysiological hyperalgesia associated to experimental inflammatory settings and, with this aim, we have performed experiments in acutely or chronically inflamed mice.

## Methods

### Animals

Swiss CD-1 male mice 7–10 week old bred in the Bioterio de la Universidad de Oviedo (Reg. 33,044 13A) on a 12-h dark–light cycle with free access to food and water were used. Experiments were performed during the light cycle in a quiet room and efforts were made to limit animal stress. All the protocols were approved by the Comité Ético de Experimentación Animal de la Universidad de Oviedo (Spain) and performed according to the guidelines of European Communities Council Directive (2010/63/EU).

### Drugs

Intraplantar (i.pl.) injections consisted in the administration of 25 µl into the right hind paw under light isoflurane (3%, Isoflo®, Esteve) anesthesia. Subcutaneous (s.c.) administration was performed under the fur of the neck in a volume of 10 ml/kg and the same volume was used for intraperitoneal (i.p.) injections. Control mice received an injection of the same volume of the corresponding vehicle or the corresponding IgG isotype when antibodies were studied.

Mouse recombinant IL-16 (Raybiotech), the non-selective COX inhibitor diclofenac (Sigma), the selective COX-1 inhibitor SC-560 (Tocris) and the selective COX-2 inhibitor celecoxib (Sigma) were dissolved in saline. The TRPV1 antagonist capsazepine (Tocris) and the TRPA1 antagonist HC030031 (Tocris) were initially dissolved in DMSO and further diluted in saline up to a maximal DMSO concentration of 10%.

The antibody against IL-16 (R&D, MAB1727) was initially reconstituted in PBS and next dissolved in saline.

### Induction of Inflammation

Acute inflammation was induced by the i.pl. injection of 25 µl of 2% type IV lambda carrageenan (Sigma) dissolved in saline into the right hind paw and assays were performed 6 h after. To evoke chronic inflammation, complete Freund´s adjuvant (CFA; Sigma) was i.pl. injected (25 µl) into the right hind paw and experiments were usually performed 4 days after although, in a particular case, behavioural assays were also performed at earlier times. Control mice were treated with sterile saline.

### Unilateral Hot Plate

As previously described (Menéndez et al. 2002), mice were gently restrained and the plantar side of the paw placed on a hot plate (IITC Life Science) set at 50ºC. Measurements of withdrawal latencies from the heated surface of each hind paw were made separately at two minute-intervals and the mean of two measures was considered. In those experiments in which treatments were systemic, means of latencies obtained separately in both paws were considered. However, in experiments in which different results in both paws were expected (after i.pl. administration or inflammation), latencies obtained in each paw were considered separately. Testing was performed during the afternoon and, for habituation, a basal measurement was done in each paw the morning of the experimental day (data not shown). Cut-off was 20 s.

### Von Frey Test

As previously reported (García-Domínguez et al. 2019), mechanical allodynia was assessed by applying von Frey filaments (Stoelting) to the plantar side of the paws. Mice were placed on the wire mesh platform and, after a 60 min period of habituation, von Frey filaments (2.44, 2.83, 3.22, 3.61, 4.08, 4.56, corresponding to a range of bending forces from 0.028 to 3.63 g) were applied. The procedure started with the 3.61 filament and six measurements, based on the ‘‘up and down’’ method (Chaplan et al. 1994), were taken in each animal randomly starting with the left or right paw. A positive response consisting in lifting, shaking, licking of the paw or marked toe spreading was followed by the application of the immediate thinner filament and the immediate thicker one was applied if the response was negative. The following formula was used to calculate the 50% g threshold: 50% response threshold = (10Xf + κδ)/10,000. Xf is the value of the last von Frey filament applied; κ is a correction factor based on pattern of responses (from the Dixon’s calibration table); δ is the mean distance in log units between stimuli (here, 0.4).

### White Cell Depletion

In depletion experiments, 50 mg/kg of cyclophosphamide (Acros Organics) dissolved in saline were i.p. injected 72 and 24 h before testing. The anti-CD4 antibody (30 μg, Biolegend 116,018, clone RM4-4) was i.p. administered in a volume of 300 μl of PBS following two different protocols. In non-inflamed mice a single dose was administered 24 h before testing. In chronically inflamed mice, since CFA inflammation is evaluated 4 days after administration, the anti-CD4 antibody was injected twice, 1 day before and 2 days after CFA. In both cases, control mice received an i.p. injection of 30 µg of the rat IgG isotype IgG2bк (eBioscience 14–4031-85). The anti-Ly6G antibody (100 μg, BioXcell BE0075-1, clone 1A8) was i.p. injected in a volume of 100 μl 18 h before in non-inflamed or carrageenan-inflamed mice and 1 day before CFA (that is, 5 days before testing) in CFA-inflamed mice. The IgG isotype used as control was, in this case, IgG2aκ (eBioscience 14–4321-85).

### Enzyme-linked Immunosorbent Assay (ELISA)

As previously described (González-Rodríguez et al. 2022), the plantar side was placed in buffer (0.1 M Tris, 0.15 M NaCl, 0.5% CTAB, Fluka) with protease inhibitor (1 tablet/10.5 ml buffer, Complete Mini Roche Diagnostics). Next, 3 µl of buffer per mg of tissue were added and tissues were homogenized with a Polytron PT 1035 (Kinematica), centrifuged (15,000 g, 15 min, 4 °C) and their supernatant protein concentration measured by using a Nanodrop 2000c (Thermo Fisher Scientific). Experiments were performed following the instructions of the manufacturer (IL-16 Duoset R&D, ELISA kit DY1727) by adding 10 µg of protein coming from homogenates or 10 µl of dialysates. Values obtained came from 6–8 independent samples assayed in duplicate.

### *In Vivo* Microdialysis

Mice receiving a i.pl. injection of carrageenan in the right hind paw and sterile saline in the left one 6 h before or i.pl. CFA in the right paw and sterile water in the contralateral one 4 days before, were anesthetized with i.p. ketamine (90 mg/kg) and xilacine (10 mg/kg). Following the previously reported procedure in rats with slight modifications (González-Rodríguez et al. 2017b), one single hollow plasmapheresis fiber (Asahi Medical Co.; 0.4 mm diameter, cut-off 3000 kDa) was inserted into the plantar subcutaneous tissue of each hind paw via a 25-gauge hypodermic needle. Fibers were perfused with saline solution via Tygon tubes (Novodirekt) attached by 25-gauge needles to 1 ml syringes which were mounted on a microdialysis pump (Syringe infusion pump 22, Harvard apparatus). After plantar passage, the fibers were inserted into Eppendorf tubes. A constant flow rate of 1 µl/min was set and dialysates were collected for 60 min. Samples were frozen at -80ºC until IL-16 determination content by ELISA.

### Flow Cytometry

When immune cells present in circulating blood were analyzed, mice were anesthetized with isoflurane (3%, Isoflo®, Zoetis) and blood was drawn by intracardiac puncture, collected in EDTA-coated tubes and incubated with red blood cell lysis buffer (150 mM NH_4_Cl, 10 mM NaHCO_3_, 1 mM EDTA, pH = 7.4; Sigma) for 10 min at 4ºC and centrifuged at 250 g for 5 min. The pellet was resuspended in 100 µl PBS containing the indicated antibodies and incubated for 20 min at room temperature (RT) in the dark. For quantification assays, a constant volume of 500 µl of peripheral blood was obtained from each individual. After labelling, samples were resuspended in 200 µl of 1 × PBS and a constant volume of 180 µl was acquired by the flow cytometer and absolute CD45^+^ cell count was determined. In CD4 depletion assays, the elimination of circulating helper T cells was verified by flow cytometry and the relative percentage of CD4 + /CD8 + T cells was assessed using anti-CD4-APC (clone GK1.5, Biolegend) and anti-CD8-APC700 (clone 53–6.7, Biolegend) monoclonal antibodies.

To study the presence of inflammatory cells in paws, footpads without tendons were cut out with scissors and a scalpel making longitudinal cuts from the ankle to the beginning of the toes prior to extraction. Tissue was chopped with scissors and scalpel into small pieces into a 35 mm Petri dish with 2 ml of digestion cocktail (Enzyme mix: Liberase™ (Roche) 300 μg/ml, DNAse1 (Sigma-Aldrich) 50 U/ml diluted in RPMI 1640 (Sigma-Aldrich) supplemented with 5% FBS (Gibco™). Samples were incubated at 37ºC for 90 min and gently shaken 1–2 times during incubation. Skin digested samples were collected into 50 ml tubes, being poured through 40 µm cell strainer that was washed with 5 ml of RPMI 1640 supplemented with 5% FBS (Broggi et al. 2016). Finally, samples were analyzed using surface and intracellular staining by flow cytometry as previously described (Sordo-Bahamonde et al. 2020). Neutrophils were identified as Ly6G^+^ cells (anti-Ly6G-APC, clone 1A8, Biolegend) by intracellular staining. In order to determine total cell numbers, samples were resuspended in 200 µl PBS, and an equal volume was acquired for each sample. For T lymphocytes analyses, samples were incubated with anti-CD3-FITC (clone 145-2C11, Biolegend), anti-CD4-APC and anti-CD8-APC700. IL-16 levels were assessed by intracellular labelling with anti-IL-16-PE (clone 14.1, Biolegend) or isotype control (clone MOPC-173, Biolegend) according to manufacturer´s protocol using BD Cytofix/Cytoperm™ Fixation/Permeabilization Kit (BD Biosciences). In order to determine total cell numbers, samples were resuspended in 200 µl PBS, and an equal volume was acquired for each sample. Data acquisition was performed using a CytoFLEX flow cytometer (Beckman Coulter) and analyzed using CytExpert 2.3 software.

### Histological and Immunofluorescence Assays

Control and inflamed mice paws were fixed overnight in 4% paraformaldehyde (Applichem GmbH; #A3813,0500) in 0.1 M phosphate buffer (pH = 7.4) at 4 °C. Subsequently, paws were paraffin-embedded, and 5 µm sections were obtained. All samples were previously heated at 65 oC for 1 h to enhance adhesion to the slide. Slides were deparaffined and hydrated by submersion in progressive alcohol-decreasing concentration solutions until distilled water. For hematoxylin–eosin staining, paws were immersed for 10 min with hematoxylin (DAKO; #GC80811-2) at RT, washed with tap water, and plunged in eosin (Sigma-Aldrich, Merck; #15,935) for 2 min at RT. Finally, samples were dehydrated and mounted using Eukitt (ORSAtec GmbH; #6,272,068) mounting medium.

For immunofluorescence assays, samples were deparaffined and hydrated as described above. For antigen retrieval, slides were heated at 95 oC for 30 min in 10 mM of sodium citrate (Sigma-Aldrich, Merck; #71,365) buffer (pH = 6.0) in a PT link system (Agilent technologies). Samples were cooled at RT and permeabilized with 0.1% Tween-20 in phosphate buffer saline (PBS; pH = 7.4) for 20 min. Next, sections were blocked with 0.15% goat serum in PBS for 1 h at RT. Subsequently, paws were incubated overnight in a humidity chamber at 4 oC with a polyclonal anti-IL-16 antibody (Invitrogen, PA5-82,627; 1:200). For double immunofluorescence assays, the Ly6G (Bio X Cell, BE0075-1; 1:200) primary antibody was also added.

The following day, samples were washed three times with PBS and incubated for 2 h at RT with the specific secondary antibodies Alexa fluor 488 goat anti rabbit (Invitrogen A-11008; 1:250) or Alexa fluor 546 goat anti rat (Invitrogen A-11081; 1:500). Paws were washed three times with PBS and counterstained with 1 µg/mL DAPI [2-(4-aminophenyl)-1H-indole-6-carboxamidine] (Invitrogen; #D1306) for 10 min at RT. Samples were again washed three times with PBS and mounted with FluoromountTM mounting medium (Sigma; F4680).

Immunostaining was observed in a Nikon Eclipse 80i epifluorescence microscope using specific filters for FITC (λex 465–495 nm; λem 515–555 nm; DM505), for G2A (λex: 510–560; λem: 590; DM575), and for DAPI counterstaining (λex 340–380 nm; λem 435–485 nm, DM400).

### Statistical Analysis

All statistical analyses were performed using GraphPad Prism software, version 6. Mean values and their corresponding standard errors were calculated. For data normally distributed, the Student’s t test was used to compare means of two groups as the number of white blood cells in mice treated with saline or cyclophosphamide or the IL-16 values in the ELISA assays in inflamed vs non inflamed paws. When more than two groups were compared, an initial one-way ANOVA analysis was performed when only one variable was considered or a two-way ANOVA was applied when two variables were considered such as inflamed and non-inflamed paws. These were followed by the Tukey’s post hoc test to establish differences. Data not normally distributed such as those obtained by the von Frey test were initially analysed by the Kruskall-Wallis test and the differences further established by the Dunn’s test. Statistical significance was set at *p* < 0.05.

## Results

### The Systemic Administration of IL-16 to Non-inflamed Mice Evoked Thermal Hyperalgesia Through the Activation of CD4+ White Blood Cells

**Fig. 1:**
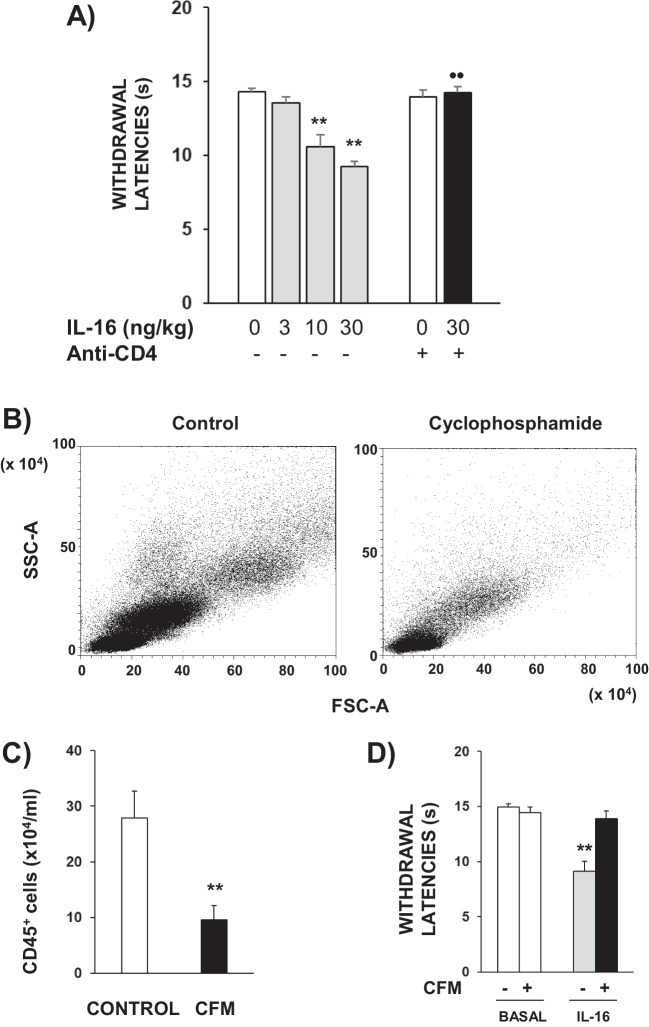
**A** Hyperalgesic effect induced by the s.c. administration of IL-16 (3–30 ng/kg, 1 h before testing) measured by the unilateral hot plate test and its prevention by the acute administration of an anti-CD4 antibody (s.c., 1 µg/kg, 1 h before). Since this effect was bilateral, the mean of latencies measured in both the right and left hind paws was taken. Means and the corresponding standard errors are represented (*n* = 6 per group). ** *p* < 0.01 compared with the control group, •• *p* < 0.01 compared with the group treated with IL-16 (30 ng/kg), Tukey’s test. **B** Representative dot plot showing the depletion of white blood cells in plasma samples from mice treated with saline (control) or cyclophosphamide (50 mg/kg; 24 and 72 h before). The dot plot displays forward scatter (FSC) on the x-axis and side scatter (SSC) on the y-axis, which represent cell size and granularity, respectively. The regions of the plot indicate different cell populations, including lymphocytes, monocytes and granulocytes. A decrease of white blood cells density is observed upon cyclophosphamide treatment. **C** Number of CD45^+^ cells per ml of blood coming from control or cyclophosphamide (CFM)-treated mice. Means and the corresponding standard errors are represented (*n* = 6 per group). ** *p* < 0.01 compared to control, Student’s t test. **D** Effect of cyclophosphamide (CFM) on the hyperalgesia induced by IL-16 (30 ng/kg, 1 h before testing). Mice received cyclophosphamide or saline 72 and 24 h and withdrawal latencies were taken before any further treatment (BASAL). Next, mice received the acute administration of IL-16 and 1 h later withdrawal latencies were measured again (*n* = 7–8 per group). Means and the corresponding standard errors are represented ** *P* < 0.01 compared with the corresponding basal latencies, Tukey’s test

In accordance to a previous report (Aguirre et al. 2020), mice receiving the systemic administration of IL-16 (3–30 ng/kg, s.c., 1 h before testing) showed thermal hyperalgesia measured by the unilateral hot plate test. Since the effect evoked by systemic IL-16 appears in both hind paws, in this particular experiment the mean of withdrawal latencies measured in both hind paws of each mouse was considered. As shown in Fig. 1A, control mice treated with saline 1 h before testing exhibited thermal withdrawal latencies close to 14 s, whereas values obtained in mice treated with increasing doses of IL-16 progressively diminished up to approximately 8–9 s after receiving the maximal dose assayed (30 ng/kg). The acute administration of an antibody against CD4 (1 µg/kg, s.c. 60 min before testing) provoked the complete prevention of the hyperalgesia induced by 30 ng/kg of IL-16 (Fig. 1A).

**Fig. 2:**
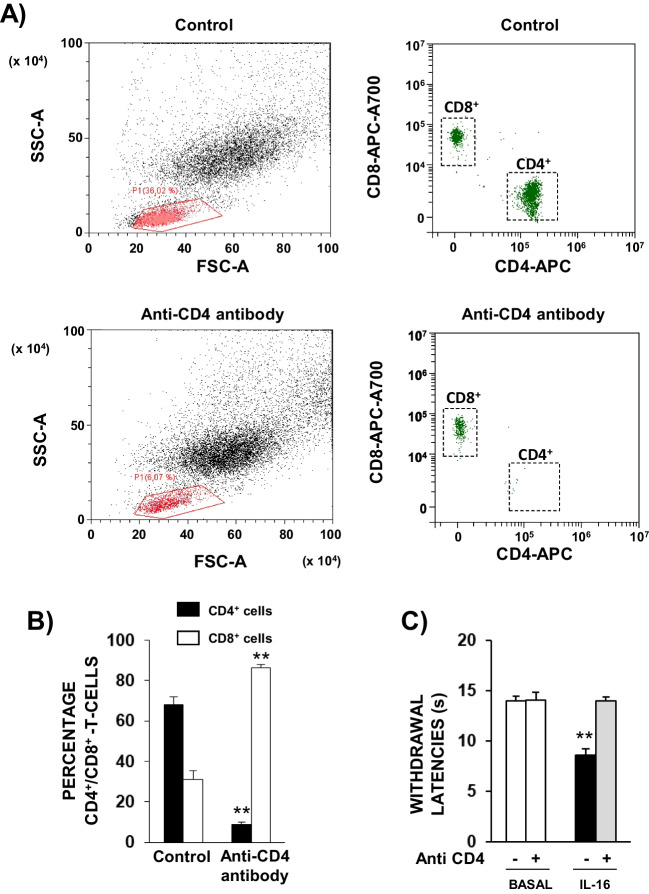
**A** Representative dot plots and gating strategy of flow cytometry assays performed on blood samples from mice treated 24 h earlier with IgG2bk (control) or 30 μg of anti-CD4 antibody. The left panels show forward scatter (FSC-A) on the x-axis and side scatter (SSC-A) on the y-axis, indicating cell size and granularity, respectively. The gated area in red represents lymphocyte population, which shows a reduction in number following anti-CD4 antibody treatment. The right panels display CD4-APC on the x-axis and CD8-APC-A700 on the y-axis, identifying CD4 + and CD8 + T cells. The decrease in CD4 + cells can be observed in the anti-CD4 antibody-treated group compared to the control. FSC: Forward scatter; SSC: Side scatter; APC: Allophycocyanin; APC-A700: Allophycocyanin-Alexa Fluor 700. **B** Percentages of circulating CD4^+^ and CD8^+^ T cells obtained by flow cytometry in mice treated with IgG2bκ isotype (control) or anti-CD4 antibody 24 h before. Means and corresponding standard errors are represented (*n* = 6 per group). ** *P* < 0.01 compared with the corresponding control values obtained in isotype-treated mice, Tukey’s test. **C** Effect induced by the depletion of CD4 with an anti-CD4 antibody on the hyperalgesia induced by IL-16. Mice received 24 h before 30 µg of an anti-CD4 antibody or IgG2bκ and withdrawal latencies were taken before any further treatment (Basal). Next, mice received the acute administration of IL-16 (30 ng/kg) and 1 h later withdrawal latencies were measured again. Means and the corresponding standard errors are represented (*n* = 6 per group). ** *P* < 0.01 compared with the corresponding basal latencies, Tukey’s test

Considering the prevalent expression of CD4 receptors in different types of immune cells, we aimed to check whether circulating white blood cells could participate in IL-16-induced hyperalgesia. The i.p. administration of 50 mg/kg of the immunosuppressant agent cyclophosphamide 72 and 24 h before testing produced a dramatic reduction in the number of total circulating white blood cells measured by flow cytometry (Fig. 1B). The quantification performed by using a CD45 antibody demonstrated that the treatment with cyclophosphamide reduced the population of leukocytes by 60% approximately related to control mice (Fig. 1C). As shown in Fig. 1D, the hyperalgesic response produced by 30 ng/kg of IL-16 was completely absent in these cyclophosphamide-depleted mice. In order to achieve the selective depletion of CD4^+^ leukocytes, mice were treated with a high dose of an anti-CD4 antibody (30 µg, 24 h before). As expected, this treatment importantly depleted circulating CD4^+^ cells (Fig. 2A-B). In contrast, the number of CD8^+^-lymphocytes remained almost unaffected (Fig. 2A) and the increased percentage of CD8^+^ lymphocytes among total T-cells shown in Fig. 2B is only a consequence of the reduction of CD4^+^ population. Supporting the relevance of CD4^+^ cells in IL-16-induced hyperalgesia, a complete suppression of the hyperalgesic effect evoked by 30 ng/kg of IL-16 was observed after CD4^+^ depletion (Fig. 2C).

### Involvement of Cyclooxygenase (COX) and TRP Channels in the Hyperalgesia Evoked by Systemic IL-16. Induction of Local Hyperalgesia After Intraplantar Administration of IL-16 to Non-inflamed Mice

**Fig. 3:**
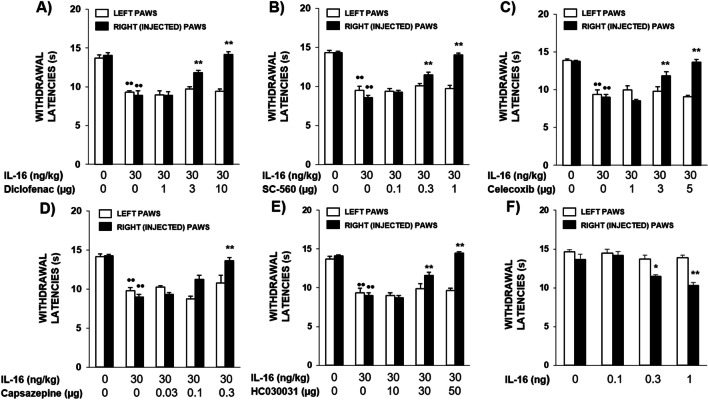
**A-E** Local inhibition of the hyperalgesia evoked by IL-16 (30 ng/kg, s.c., 1 h before testing) following the i.pl administration into the right paw (black bars) of the non-selective cyclooxygenase (COX) inhibitor diclofenac (**A**; 1–10 µg, 1 h before testing), the selective COX-1 inhibitor SC-560 (**B**; 0.1–1 µg, 1 h before testing,), the selective COX-2 inhibitor celecoxib (**C**; 1–5 µg, 1 h before testing), the TRPV1 channel inhibitor capsazepine (**D**; 0.03–0.3 µg, 1 h before testing) or the TRPA1 channel inhibitor HC030031 (**E**; 10–50 µg, 1 h before testing). Means and corresponding standard errors are represented (*n* = 5–7 per group). •• *p* < 0.01 compared with the control group, ** p < 0.01 compared with the group treated with IL-16 (30 ng/kg), Tukey’s test. **F**) Local hyperalgesic effect induced after the i.pl. administration of IL-16 (0.1–1 ng, 1 h before testing) in the injected paws (right, black bars) measured by the unilateral hot plate test. Means and corresponding standard errors are represented (*n* = 6 per group). * *p* < 0.05, ** *p* < 0.01 compared with the control group, Tukey’s test

The hyperalgesia produced after s.c. administration of 30 ng/kg of IL-16 1 h before testing was dose-dependently and locally inhibited after the i.pl. administration into the right paw of either the non-selective COX inhibitor diclofenac (1–10 µg, 1 h before, Fig. 3A), the COX-1 selective inhibitor SC-560 (0.1–1 µg, 1 h before, Fig. 3B) or the COX-2 selective inhibitor celecoxib (1–5 µg, 1 h before, Fig. 3C). Since in this experiment inhibitory drugs were locally administered in only one paw, latencies obtained in left and right paws were analysed separately. None of these drugs modified withdrawal latencies when administered alone at the highest dose studied (not shown). Furthermore, IL-16-induced hyperalgesia was also locally inhibited after the i.pl. administration of the selective TRPV1 channels blocker capsazepine (0.03–0.3 µg, 1 h before, Fig. 3D) or the selective TRPA1 channel blocker HC030031 (10–50 µg, 1 h before, Fig. 3E), thus supporting the involvement of TRP channel sensitization in this effect.

The fact that the i.pl. administration of COX inhibitors or TRP channel blockers locally prevents the hyperalgesia evoked by systemic IL-16 in the paw injected with the inhibitor strongly supported the involvement of local mechanisms in its effect. To check this possibility, we assessed if the direct i.pl. administration of IL-16 could provoke a hyperalgesic reaction limited to the injected paw. Effectively, the injection of 0.1–1 ng of IL-16 into the right paws of mice 1 h before testing, produced dose-dependent hyperalgesia in the injected paw whereas latencies measured in the contralateral one remained unaltered (Fig. 3F).

### The Development of Acute or Chronic Inflammation Evokes a Local Increase in IL-16 Concentration Mainly Related to Neutrophil Infiltration

**Fig. 4:**
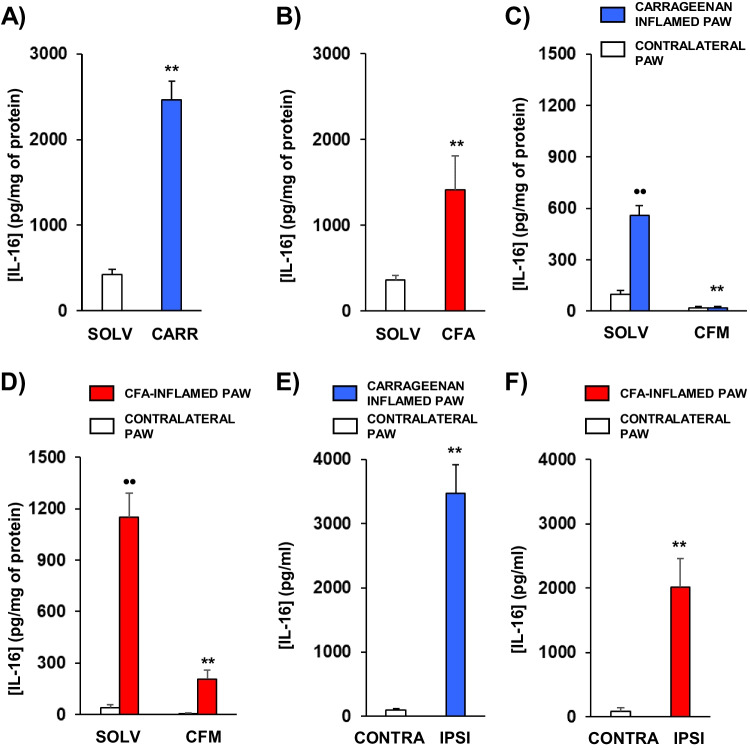
**A** IL-16 levels measured by ELISA in homogenates coming from paws acutely inflamed after the injection of carrageenan (CARR; 25 µl, 6 h before) or receiving sterile saline (SOLV; 25 µl, 6 h before). **B** IL-16 levels measured by ELISA in homogenates coming from paws chronically inflamed after the injection of CFA (25 µl, 96 h before) or sterile water (SOLV; 25 µl, 96 h before). Means and corresponding standard errors are represented (n = 8 per group). ** *p* < 0.01 compared with solvent-treated group, Student’s t test. **C** Levels of IL-16 measured by ELISA in homogenates of paws acutely inflamed by the i.pl. administration of carrageenan and of their contralateral non-inflamed ones coming from mice receiving the administration of cyclophosphamide (CFM; 50 mg/kg i.p., 72 and 24 h before) or the same volume of saline (SOLV). **D** Levels of IL-16 measured by ELISA in homogenates of paws chronically inflamed by the i.pl. administration of CFA and in their contralateral non-injected ones in mice receiving cyclophosphamide (CFM) or sterile water (SOLV). Means and corresponding standard errors are represented (*n* = 6–7 per group). •• *p* < 0.01 compared with the contralateral paw, ** *p* < 0.01 compared with the inflamed paw of solvent-treated group, Tukey’s test. **E** Concentration of IL-16 measured by ELISA in microdialysates of paws acutely inflamed after the injection of carrageenan (IPSI; 25 µl, 6 h before) or sterile saline in the contralateral paw (CONTRA; 25 µl, 6 h before). **F** Concentration of IL-16 measured by ELISA in microdialysates of paws chronically inflamed after the injection of CFA (IPSI; 25 µl, 96 h before) or receiving sterile water in the contralateral paw (CONTRA; 25 µl, 96 h before). Means and their corresponding standard errors are represented (*n* = 5 per group). ** *p* < 0.01 compared with the contralateral paw, Student’s t test

The induction of local hyperalgesia after the i.pl. administration of IL-16 opened the possibility that this cytokine could act as a mediator involved in the hyperalgesic responses linked to tissue injury. We initially studied whether the presence of IL-16 could be increased in tissues submitted to local processes that, as inflammation, are associated to hyperalgesic responses. ELISA assays were performed in paw homogenates coming from mice with acute or chronic inflammation due to the injection of carrageenan or CFA, respectively. As shown in Fig. 4, IL-16 concentration measured in inflamed paws was in both cases significantly higher than that found in paws coming from control mice treated with the corresponding vehicle. Thus, paws acutely inflamed by carrageenan showed an approximately six-fold increase related to controls (Fig. 4A) whereas chronic inflammation evoked by CFA treatment 96 h before led to a four-fold augmentation of IL-16 levels (Fig. 4B). In order to explore the relevance of immune cells in this increase of IL-16 levels during inflammation, we performed an assay after leukocyte depletion with cyclophosphamide. Showing the outstanding role played by white blood cells, their depletion almost totally prevented the increase of IL-16 concentration observed in both types of inflammatory processes (Fig. 4C-D). Finally, when IL-16 levels were measured in the extracellular liquid present in the inflammatory milieu obtained by microdialysis, a remarkable increase of IL-16 concentration was also detected both in carrageenan- (Fig. 4E) and CFA-inflamed (Fig. 4F) paws.

**Fig. 5:**
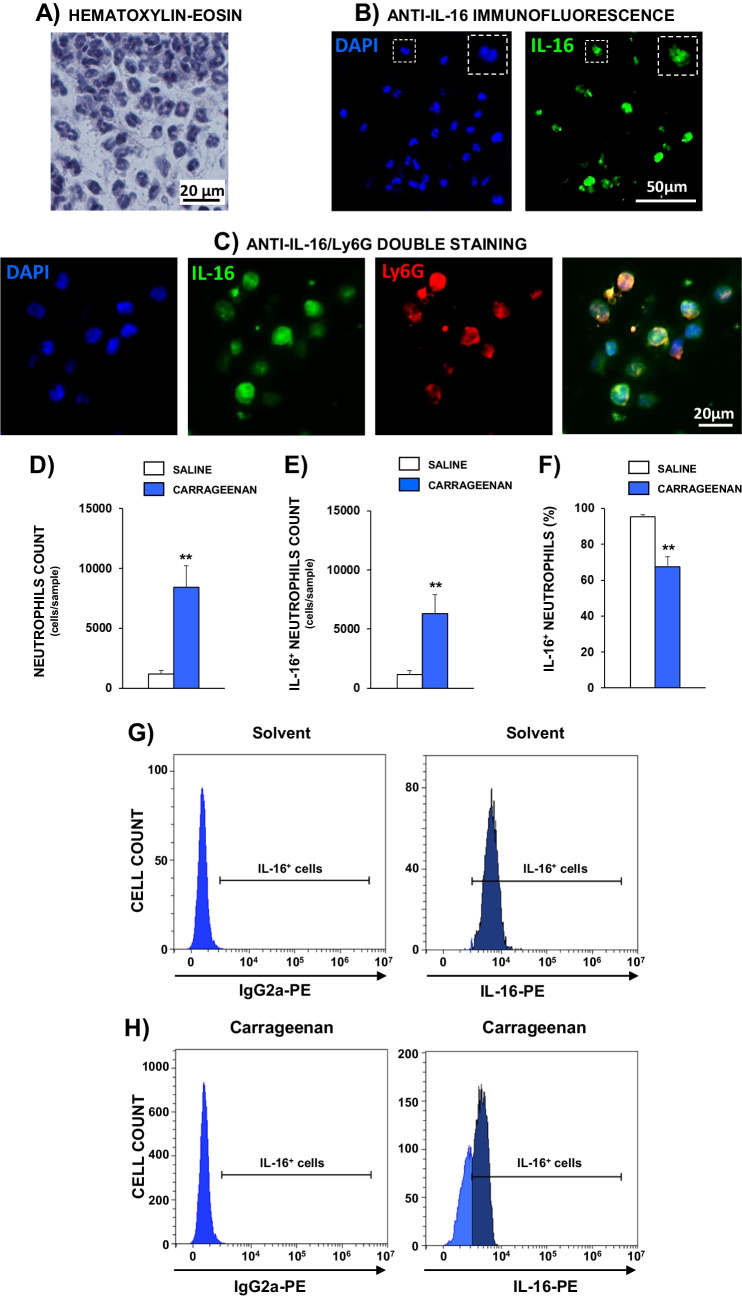
Microphotographs obtained from hind paw plantar tissues of mice treated i.pl. with carrageenan (25 µl, 6 h before). **A** Microphotographs corresponding to hematoxylin–eosin staining. Images were taken at 1000 × of original magnification. **B** Cell nuclei stained with DAPI (blue, upper left image) and IL-16^+^ cells labelled with an anti-IL-16 antibody (green, upper right photograph). Images were taken at 400 × of original magnification. **C** Staining of cells with DAPI (blue, left side), immunolabelling with anti-IL-16 antibody (green), anti-Ly6G antibody (red) and merged image of double staining with both antibodies (right side). Images were taken at 1000 × of original magnification. In graphs D-H, results obtained by flow cytometry in cells coming from paw plantar tissues are shown. **D** Total number of neutrophils (Ly6G + cells) obtained from saline- or carrageenan-treated paws. **E** Number of neutrophils that express IL-16 (Ly6G + IL-16 + cells) obtained from saline- or carrageenan-treated paws. **F** Percentage of neutrophils that express IL-16 (Ly6G + IL-16 + cells) in relation to the total number of neutrophils (Ly6G + cells) obtained from saline- or carrageenan-treated paws. Graphs represent the mean and the corresponding standard error (*n* = 5–6 per group). In (**G**) and (**H**), representative histograms of IL-16 expression on neutrophils in solvent- (**G**) or carrageenan-treated mice (**H**) are shown. PE: Phycoerythrin

Trying to determine the main immune subsets responsible for IL-16 expression in inflamed tissues, immunofluorescence assays were performed in carrageenan- and CFA-treated paws. In carrageenan-inflamed paws, an initial morphological analysis of hematoxylin–eosin stained samples suggested that most of the immune cells present were probably neutrophils (Fig. 5A), whereas the number of lymphocytes and macrophages observed was much lower. Furthermore, as illustrated in Fig. 5B, immunofluorescence assays performed with an anti-IL16 antibody revealed the presence of an important number of IL-16^+^ inflammatory cells in paws acutely inflamed with carrageenan. In accordance with both findings, double immunofluorescence assays performed with an anti-IL-16 antibody and an anti-Ly6G antibody addressed against neutrophils confirmed the important infiltration of neutrophils expressing IL-16 in carrageenan-inflamed paws (Fig. 5C). Flow cytometry analysis confirmed the information obtained by immunofluorescence assays showing that, whereas the presence of neutrophils is very scarce in vehicle-treated paws, a remarkable number of these immune cells marked with the anti-Ly6G antibody appears in inflamed paws (Fig. 5D). In agreement with immunofluorescence assays, most of these Ly6G^+^ cells present in carrageenan-inflamed paws also expressed IL-16, being the number of IL-16^+^ neutrophils present in inflamed paws about 8 times greater than in control ones (Fig. 5E). Another interesting finding obtained by flow cytometry experiments was related to the comparison percentage of IL-16^+^ neutrophils present in non-inflamed and carrageenan-inflamed paws. Thus, about 95% of the scarce number of neutrophils detected in control paws was IL-16^+^ whereas this percentage was about 67% in carrageenan-inflamed paws, suggesting that the resting 33% have probably released its initial content of IL-16 (Fig. 5F-H).

**Fig. 6:**
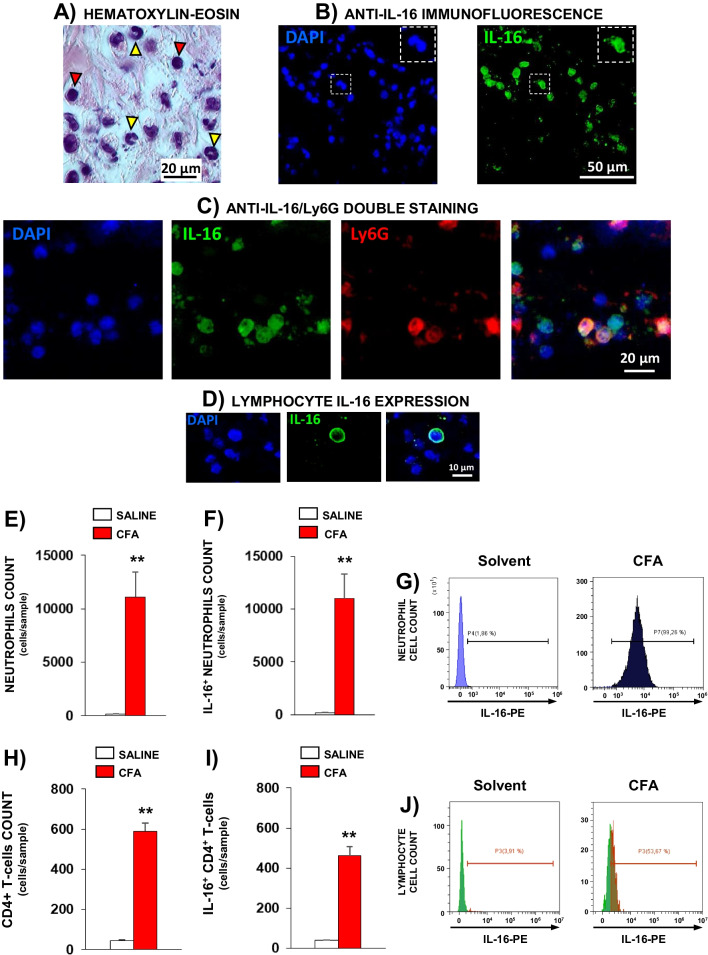
Microphotographs obtained from hind paw plantar tissues of mice treated i.pl. with CFA (25 µl, 96 h before). **A** Microphotographs corresponding to hematoxylin–eosin staining. Images were taken at 1000 × of original magnification. Representative examples of neutrophils or lymphocytes are indicated by yellow or red triangles, respectively. **B** Cell nuclei stained with DAPI (blue, upper left image) and IL-16^+^ cells stained with an anti-IL-16 antibody (green, upper right photograph). Images were taken at 400 × of original magnification. **C** Staining of cells with DAPI (blue, left side), immunolabelling with anti-IL-16 antibody (green), anti-Ly6G antibody (red) and merged image of double labelling with both antibodies (right side). Images were taken at 1000 × of magnification. **D** Image of the staining with the anti-IL-16 antibody of one immune cell with the characteristic nucleus shape of lymphocytes 1000 × of original magnification. **E-J** Determination by flow cytometry of the number IL-16^+^ neutrophils and IL-16^+^-CD4^+^-lymphocytes in solvent-treated or CFA-inflamed paws. **E** Mean and the corresponding standard error (*n* = 5–6 per group) of the number of neutrophils. **F** Mean and the corresponding standard error of the number IL-16^+^ neutrophils. **G** Representative histograms of IL-16 expression on neutrophils in solvent- or CFA-treated mice. **H** Mean and the corresponding standard error (*n* = 5–6 per group) of the number of CD4^+^-lymphocytes. **I** Mean and the corresponding standard error of the number IL-16^+^-CD4^+^-lymphocytes. **J** Representative histograms of IL-16 expression on CD4^+^-lymphocytes in solvent- or CFA-treated mice. PE: Phycoerythrin

Related to CFA inflammation, histological analysis of hematoxylin–eosin stained samples showed that neutrophils were practically absent in controls treated with vehicle 4 days before, thus impeding the evaluation of IL-16 expression in them. In contrast, an important presence of neutrophils was observed in CFA-inflamed paws that were accompanied by a reduced number of cells with a lymphocyte-like nuclear morphology (Fig. 6A) whereas, as before, the presence of macrophages was very scarce.

As occurred in carrageenan-inflamed paws, IL-16^+^ cells were observed by immunofluorescence assays performed in paws inflamed with CFA (Fig. 6B) and double immunohistochemical experiments with the anti-IL-16 antibody accompanied by an anti-Ly6G antibody again demonstrated the profuse presence of neutrophils containing IL-16 (Figs.6C). In addition, the expression of IL-16 was also detected in cells morphologically identified as lymphocytes (Fig. 6D). This view was confirmed by flow cytometry experiments in which a remarkable increase of Ly6G^+^ cells was detected in the inflamed paws (Fig. 6E), being most of them IL-16^+^ (Fig. 6F). Besides, CD3^+^ lymphocytes constituted about 10% of cells and the identification of T-cells subsets revealed that most of them were CD4^+^ whereas CD3^+^-CD8^+^ were almost completely absent. Moreover, the detection of CD3^+^ cells in non-inflamed paws was, as expected, extremely infrequent (about 45 cells/paw; Fig. 6H), hindering IL-16 levels evaluation. Finally, it seems remarkable that, contrasting with the observation made in carrageenan-inflamed mice, in this case almost all neutrophils present in CFA-inflamed paws were IL-16^+^ (Fig. 6G) whereas a small percentage of CD4^+^ cells did not show IL-16 expression (Fig. 6J).

**Fig. 7:**
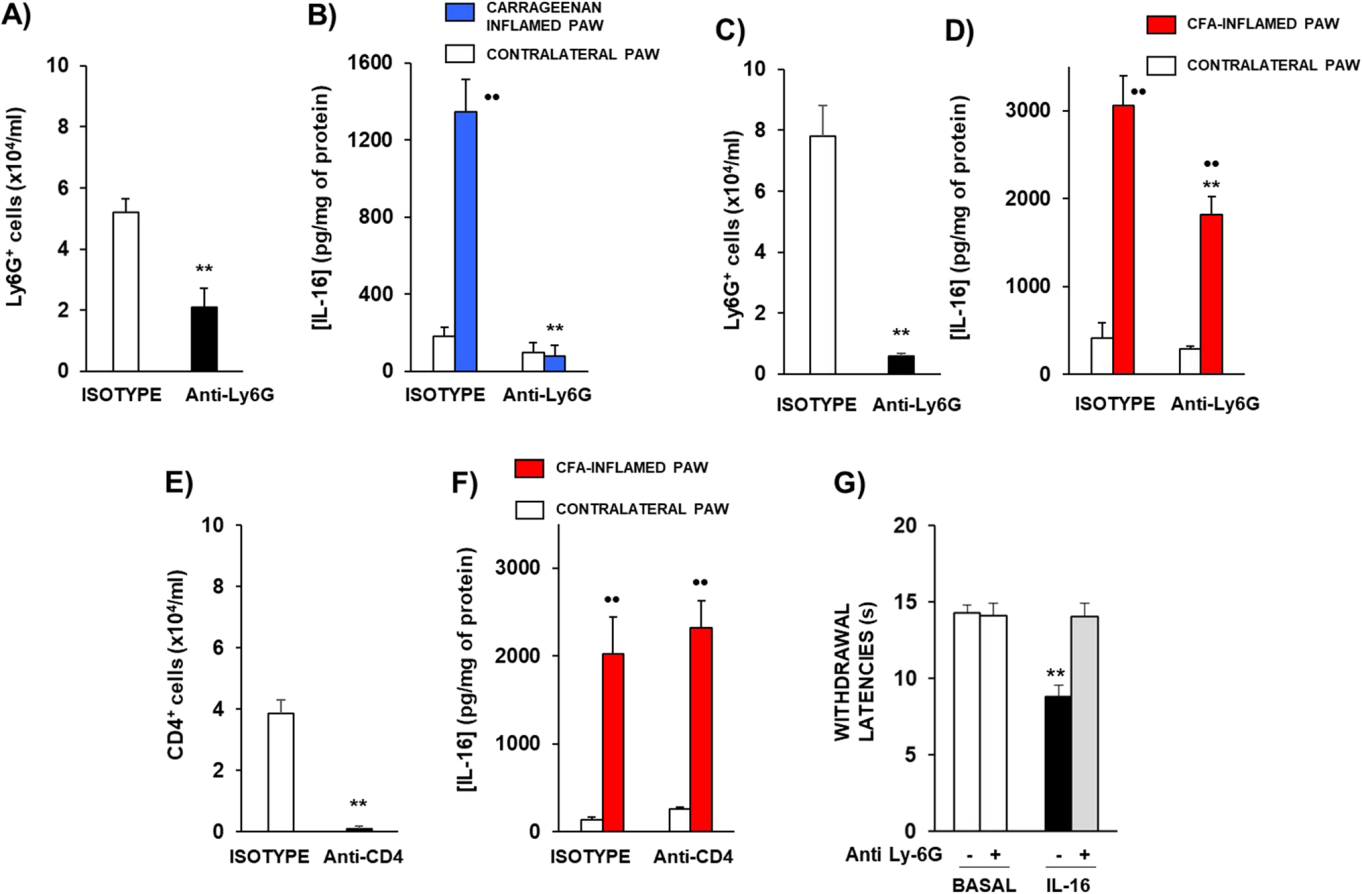
**A** Number of Ly6G + cells measured by flow cytometry in blood coming from mice treated with isotype IgG2aκ or anti-Ly6G antibody (i.p.; 100 µg; 18 h before). **B** Levels of IL-16 measured by ELISA in homogenates of paws acutely inflamed by the i.pl. administration of carrageenan 6 h before and of their contralateral ones treated with saline, coming from mice receiving the administration of isotype IgG2aκ or anti-Ly6G antibody (i.p.; 100 µg; 18 h before). **C** Number of Ly6G + cells measured by flow cytometry in blood coming from mice treated with isotype IgG2aκ or anti-Ly6G antibody (i.p.; 100 µg; 5 days before). **D** Levels of IL-16 measured by ELISA in homogenates of paws inflamed by the i.pl. administration of CFA and of their contralateral non-inflamed ones coming from mice receiving the administration of isotype IgG2aκ or anti-Ly6G antibody (i.p.; 100 µg; 5 days before). **E** Number of CD4 + cells measured by flow cytometry in blood coming from mice treated with isotype IgG2bκ or anti-CD4 antibody (i.p.; 30 µg; 5 and 3 days before testing). **F** Levels of IL-16 measured by ELISA in homogenates of paws inflamed by the i.pl. administration of CFA and of their contralateral non-inflamed ones coming from mice receiving the administration of anti-CD4 antibody (i.p.; 30 µg; 5 and 3 days before testing, that is 1 day before and 2 days after CFA administration) or isotype IgG2bκ. **G** Effect of neutrophil depletion induced by the administration of an anti-Ly6G antibody on the hyperalgesia induced by IL-16. Mice received the i.p. administration of 100 µg of the anti-Ly6G antibody or IgG2bκ isotype 18 h before and withdrawal latencies were taken before any further treatment (Basal). Next, mice received the acute administration of IL-16 (30 ng/kg) and 1 h later withdrawal latencies were measured again. Means and their corresponding standard errors are represented (*n* = 6-10 per group). In A, C, and E: ** *p* < 0.01 compared to isotype-treated group, Student´s t test. In B, D, F: •• *p* < 0.01 compared to the corresponding contralateral paw; ** *p* < 0.01 compared to inflamed paw of mice treated with isotype, Tukey’s test. In G, ** *p* < 0.01 compared to the corresponding basal value, Tukey’s test.

These results show that neutrophils are probably the main line of immune cells involved in the augmentation of IL-16 observed in inflamed territories. Apart from this, their abundant presence in inflamed tissues together with the scarce presence of lymphocytes, especially in carrageenan-inflamed mice, raises the possibility that neutrophils could also be a relevant cellular target for IL-16 to evoke hyperalgesia. In order to further check these two possibilities, experiments were performed in mice in which neutrophils were depleted. The i.p. treatment with 100 µg/mouse of anti-Ly6G antibody 18 h before testing significantly reduced the number of circulating Ly6G + cells measured by flow cytometry assays (Fig. 7A). Interestingly, this effect was accompanied by the complete prevention of the IL-16 concentration increase in carrageenan-inflamed paws (Fig. 7B). Related to chronic inflammation, in order to evoke the reduction of neutrophil population during the 4 days necessary for CFA-induced inflammation to develop, mice received 100 µg of the anti-Ly6G antibody the day before CFA administration, that is, 5 days before testing. As occurred when administered 18 h before, this schedule also evoked a remarkable reduction in circulating Ly6G^+^ cells (Fig. 7C) that was accompanied by a significant, although not complete, decrease of IL-16 concentration measured in CFA-inflamed mice (Fig. 7D). Since the efficacy of neutrophil depletion was only partial in CFA-inflamed mice and the presence of lymphocytes was larger than in carrageenan-inflamed paws, we checked whether lymphocytes could also be involved in IL-16 up-regulation in this model. Mice received an initial i.p. injection of 30 µg of the anti-CD4 antibody the day before CFA administration and a second one 2 days after CFA. As shown in Fig. 7E, this treatment evoked an important reduction in circulating CD4^+^ cells although the increase of IL-16 concentration observed in CFA-inflamed paws remained unaltered (Fig. 7F), thus indicating that these cells do not importantly contribute to the augmented presence of IL-16 in CFA-inflamed paws.

Finally, since our results demonstrate that neutrophils are the most important population of immune cells present in inflamed tissues, we wondered whether they could not exclusively act as a source of IL-16 but also as targets for IL-16 to exert its effects. Thus, we have studied whether the hyperalgesic effect evoked by IL-16 could be affected following neutrophil depletion. As shown in Fig. 7G, the hyperalgesic effect evoked after the exogenous administration of 30 ng/kg of IL-16 was completely absent in mice in which Ly6G + cells were depleted by the administration of 100 µg of the anti-Ly6G antibody.

### Antinociceptive Effect Evoked by the Administration of an anti-IL-16 Antibody on Thermal Hyperalgesia and Mechanical Allodynia Measured in Acutely or Chronically Inflamed Mice

**Fig. 8:**
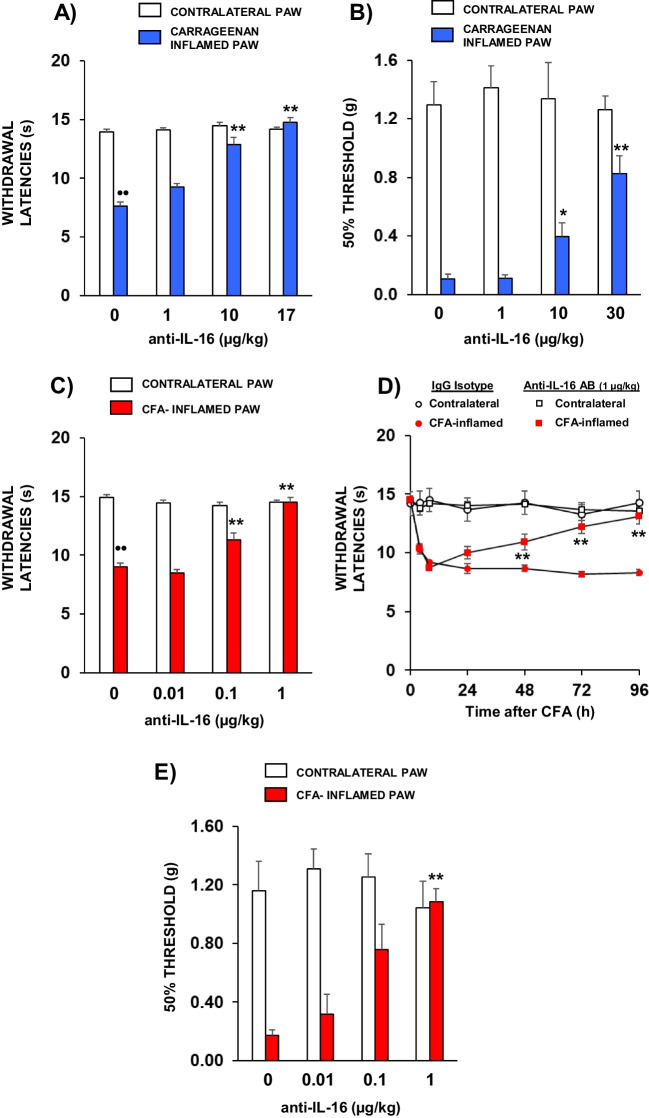
The administration of an anti-IL-16 antibody (s.c., 1 h before testing) dose-dependently inhibited hypernociceptive responses evoked in mice by the i.pl. administration of carrageenan 6 h before or CFA 96 h before. **A** Inhibition of carrageenan-induced thermal hyperalgesia measured by the unilateral hot plate test in response to the administration of the anti-IL-16 antibody. **B** Inhibition of carrageenan-induced mechanical allodynia measured by the von Frey test in response to the administration of the anti-IL-16 antibody. The administration of the maximal dose of the anti-IL16 antibody or the corresponding IgG isotype did not evoke any effect when administered alone, although these data are not represented for clarity. **C** Inhibition of CFA-induced thermal hyperalgesia measured by the unilateral hot plate test in response to the administration of the anti-IL-16 antibody. D) Effect of the administration of 1 µg/kg of the anti-IL-16 antibody (s.c., 1 h before testing) or the corresponding IgG2bк isotype on the hyperalgesic effect evoked at different times (4 h-4 days) after CFA injection. E) Inhibition of CFA-induced mechanical allodynia measured by the von Frey test in response to the administration of the anti-IL-16 antibody. Means and their corresponding standard errors are represented (*n* = 5–9 per group). •• *p* < 0.01 compared to their contralateral paw in mice treated with IgG2bκ, ** *p* < 0.01 compared to inflamed paws of mice treated with IgG2bκ, Tukey’s test (**A, C, D**). •• *p* < 0.01 compared to their contralateral paw in mice treated with IgG2bκ, ** *p* < 0.01 compared to inflamed paw of mice treated with IgG2bκ, Dunn’s test (**B, E**)

The potential role played by IL-16 in acute inflammatory hypernociception was assessed by administering an anti-IL-16 antibody to carrageenan-inflamed mice. As expected, carrageenan-induced inflammation provoked a marked reduction of nociceptive withdrawal latencies measured by the unilateral hot plate test. The s.c. administration of the anti-IL-16 antibody (1–17 µg/kg, 1 h before testing) dose-dependently reduced carrageenan thermal hyperalgesia, being withdrawal latencies completely undistinguishable from those measured in contralateral paws when the maximal dose was assayed (Fig. 8A).

Mechanical allodynia was assessed by using von Frey filaments. The i.pl. administration of carrageenan reduced 50% paw mechanical threshold in mice treated with IgG2bк control isotype from a 50% threshold of about 1 g in contralateral, non-inflamed, paws up to values in the range 0.1 g in paws inflamed with carrageenan. The s.c. administration of the anti-IL-16 antibody (1–30 µg/kg, 1 h before) provoked a dose-dependent increase of 50% withdrawal threshold of inflamed paws that was significant for the dose of 10 µg/kg and produced a 7 times higher threshold in mice receiving 30 µg/kg of the anti-IL-16 antibody than that obtained in inflamed control mice treated with the isotype IgG2bк (Fig. 8B). In any case, the inhibition of mechanical allodynia was not complete, even for doses of the anti-IL-16 antibody higher than those able to totally revert thermal hyperalgesia.

The role played by IL-16 in the hyperalgesia derived from chronic inflammation was assessed in mice treated with i.pl. CFA 96 h before. Mice inflamed with CFA receiving the s.c. administration of the IgG2bк isotype showed thermal hyperalgesia, whereas this effect was dose-dependent inhibited in animals treated with anti-IL16 antibody at doses lower than those effective in carrageenan-inflamed mice. Thus, the s.c. administration of 0.1 µg/kg of the anti-IL-16 antibody 1 h before testing, already produced a significant inhibition of CFA-induced hyperalgesia whilst the dose of 1 µg/kg completely reverted it (Fig. 8C). To directly assess whether the difference between the effective dose range of the IL-16 antibody when assayed in carrageenan- or CFA-inflamed mice was due to a greater potency in chronic than acute inflammatory settings, we performed an experiment in which we measured the effect of a unique dose of the anti-IL-16 antibody (1 µg/kg) assayed at different times after CFA inoculation (4, 8, 24, 48, 72 and 96 h). In order to avoid the excessive repetition of treatments and measurements and to trying to minimize the use of mice, two different groups of 11 CFA-inflamed mice were included. One group of them received 1 h before testing either the administration of the isotype or the anti-IL-16 antibody and was tested at 4, 24 and 72 h after CFA administration. The same procedure was applied to other group that was tested 8, 48 and 96 h after CFA administration and, for clarity, all data obtained were represented in the same graph. As shown in Fig. 8D, significant hyperalgesia was detected as early as 4 h after CFA administration reaching a peak at 24 h that was maintained during all the period studied. Interestingly, the administration of 1 µg/kg of the anti-IL-16 antibody was ineffective 4, 8 or even 24 h after CFA injection, but a significant antihyperalgesic effect was observed 2 days after CFA administration, being maximal from day 3.

The effect of IL-16 neutralization was also assessed on chronic mechanical allodynia evoked by CFA. In this case, the s.c. administration of the anti-IL-16 antibody dose-dependently blocked CFA-induced allodynia reaching a complete inhibition after the administration of 1 µg/kg (Fig. 8E), a dose range similar to the effective one against thermal hyperalgesia.

## Discussion

The present report is addressed to characterize the hyperalgesic response produced by IL-16 in mice and to explore the possible involvement of this cytokine in pathological pain, focussing on experimental inflammatory settings. As described in a previous report (Aguirre et al. 2020), the systemic administration of IL-16 to mice evokes thermal hyperalgesia in both hind paws measured by the unilateral hot plate test. Here we show that this IL-16-induced hyperalgesia is prevented when CD4, the main target where IL-16 binds to exert its effects (Liu et al. 1999), is neutralized by the acute administration of a low dose of an anti-CD4 antibody. Since CD4 surface proteins are mainly expressed in T-lymphocytes but also in particular populations of monocytes (Graziani-Bowering and Filion 2000) or neutrophils (Biswas et al. 2003), their involvement in IL-16-induced hyperalgesia strongly supports the participation of leukocytes in this process. This possibility seems feasible considering the relevant role played by white blood cells in the hyperalgesic responses triggered by other cytokines (Yoshida et al. 2018) or chemokines (Liu et al. 2021; Llorián-Salvador et al. 2016a) and the participation of the immune system in painful responses related to different pathological settings (Liu et al. 2021; Malcangio et al. 2019). However, it should be also considered that CD4 receptors can be expressed by some types of neurons (Fenster et al. 2010; Omri et al. 1994) and that IL-16 could directly activate peripheral nociceptive neurons (Kanngiesser et al. 2012). Thus, to elucidate the relevance of leukocytes in the hyperalgesic response triggered by IL-16, we used cyclophosphamide, a molecule able to reduce the circulating population of white blood cells (Aguirre et al. 2020). Interestingly, the cyclophosphamide-induced drastic reduction in the concentration of blood leukocytes was accompanied by the complete prevention of the hyperalgesic response evoked by IL-16. Furthermore, also the selective depletion of CD4^+^ cells observed 24 h after the treatment with a high dose of the anti-CD4 antibody (González-Rodríguez et al. 2022), impedes the instauration of IL-16-induced hyperalgesia, thus demonstrating that circulating white blood cells expressing CD4 receptors are crucial for this effect.

Related to the pronociceptive mechanisms leading to IL-16 evoked hyperalgesia, our results show that the effect elicited by IL-16 is related to prostaglandin (PG) synthesis and TRPA1 and TRPV1 channel sensitization. The involvement of PG synthesis was initially deduced by the inhibitory effect observed after the administration of the COX inhibitor diclofenac and the contribution of COX-1 or COX-2 was demonstrated by using selective inhibitors of both COX isoforms. To our knowledge, no previous report has explored the possibility that IL-16 may directly evoke the release of PG. However, the fact that PG synthesis can be a final step in a cascade triggered by IL-16 seems feasible considering that IL-16 induces the production of IL-1β, IL-6 or TNFα in different tissues (Seegert et al 2001; Zhang et al. 2024), being all of them cytokines whose ability to evoke PG synthesis has been largely demonstrated (Dayer et al. 1986; Navarra et al. 1992; Kettelhut et al. 1987).

In addition, since the ability of PGs to evoke hyperalgesia is related to their ability to amplify the activity of different types of TRP channels in nociceptors (Jang et al. 2020) as TRPV1 (Moriyama et al. 2005) and TRPA1 (Dall'Acqua et al. 2014), we have explored whether the sensitization of these channels could be a final step in the effect of IL-16. Supporting this view, IL-16-evoked hyperalgesia was dose-dependently prevented by the i.pl. injection of either the TRPV1 antagonist capsazepine or the TRPA1 antagonist HC030031. In order to explain the involvement of TRPV1 and TRPA1 in IL-16-induced hyperalgesia, it seems reasonable to propose that a rapid channel phosphorylation similar to that produced by PGs on both types of TRP channels (Katsura et al. 2007) could occur. In addition, the up-regulation of TRPV1 and TRPA1 channels in response to IL-16 could also be envisaged. However, the previous description that the overexpression of TRP channels in response to PGs needs several hours or days to occur (Mistry et al 2014; Dall'Acqua et al. 2014) makes this possibility unlikely.

Both the initial description of IL-16-induced hyperalgesia (Aguirre et al. 2020) and the experiments commented in the above paragraphs have been performed after the systemic administration of IL-16. However, the observation that the local i.pl. injection of COX inhibitors or TRP blockers prevents the hyperalgesic action of systemic IL-16 uniquely in the paw receiving the administration of the inhibitor, without affecting the contralateral one, strongly suggested that IL-16 is able to activate local hyperalgesic mechanisms. Confirming this view, the direct i.pl. administration of small doses of IL-16 produced dose-dependent hyperalgesia restricted to the injected paw. This ability of IL-16 to locally amplify nociceptive responses make plausible the possibility that this interleukin could play a role in the hyperalgesia associated to tissue injury, as occurs during inflammation. Moreover, the participation of prostaglandins in IL-16 hyperalgesia fits with their recognized role in inflammatory peripheral sensitization (Jang et al. 2020). A necessary premise to make possible this proposition should be the presence of augmented IL-16 levels in inflamed environments and, accordingly, a remarkable increase in IL-16 concentration was observed by ELISA assays performed in homogenates coming from paws affected by either acute inflammation due to carrageenan administration or chronic inflammation produced by CFA. Furthermore, the almost complete prevention of this increase in IL-16 concentration in the inflamed milieu that occurs after leukocyte depletion with cyclophosphamide strongly suggests that chemotaxis of white blood cells towards the injured territory is essential in this process. Finally, ELISA assays performed in extracellular fluids obtained by microdialysis clearly indicate that the higher levels of IL-16 present in inflamed paws is related to its release to the extracellular milieu and is not due to the possible detection of its inactive precursor or to the augmentation of its concentration at intracellular level. Since the role played by IL-16 in inflammation has not been as profusely studied as that of other interleukins, we are not aware about previous studies that quantify IL-16 levels in carrageenan- or CFA-inflamed mice. However, since TNF plays a key role in the development of both carrageenan- (Rocha et al. 2006) and CFA-induced (Inglis et al. 2005) inflammation, our result fits well with the previous description of IL-16 release in response to the action of TNF on TNFR1 (Ahn et al. 2014).

Once the increased presence of IL-16 was detected in inflamed tissues, we aimed to elucidate the type of cells responsible for its expression by using immunohistochemical and flow cytometry assays. Globally, our results point towards a fundamental role for neutrophils in both inflammatory situations, although lymphocytes could also contribute during chronic inflammation. Initially, the population of immune cells present in paws acutely inflamed by carrageenan is mainly formed by neutrophils, as expected considering their basic role in rapid innate immunity responses and in accordance with previous reports (Rocha et al. 2006; Llorián-Salvador et al. 2016b). Immunofluorescence experiments showed a profuse presence of cells stained with both anti-Ly6G and anti-IL-16 antibodies in carrageenan–inflamed paws and this result was confirmed by flow cytometry analysis, further supporting that neutrophils could be the main cell line related to IL-16 expression during acute inflammation. Although initial studies suggested that IL-16 was mainly produced by CD4^+^ and CD8^+^ lymphocytes (Zhang et al. 1998; Wu et al. 1999), more recent reports have consistently described the ability of neutrophils to synthetize and release IL-16 (Roth et al. 2015, 2016; Donati et al. 2017). In fact, a possible relationship between the expression of IL-16 in neutrophils and the development of lung metastasis has been proposed in mice (Donati et al. 2017). Furthermore, the presence of cytosolic IL-16 as well as its release during secondary necrosis has been observed in human neutrophils (Roth et al. 2016). The detection of high concentrations of IL-16 in our experiments performed in microdialysates obtained in vivo from carrageenan- or CFA- inflamed paws strongly suggest that IL-16 is not only expressed by neutrophils but also released to the extracellular liquid of inflamed tissues. In fact, the presence of IL-16 in almost all neutrophils found in non-inflamed territories seems to suggest that the role played by IL-16 in inflamed tissues could be more related to its release than to de novo synthesis. In support of that, flow cytometry experiments showed that a non-negligible percentage of neutrophils present in carrageenan-inflamed paws are not labelled with the anti-IL-16 antibody. Finally, experiments in which neutrophils were depleted after treatment with an anti-Ly6G antibody confirmed that the reduction in neutrophil population importantly prevents the increase of IL-16 concentration observed in inflamed paws. Thus, neutrophil depletion completely inhibited IL-16 presence in carrageenan-inflamed paws and evoked a significant, although not complete, reduction in CFA-inflamed paws. This partial effect and the previous finding that the presence of lymphocytes is greater in the CFA model could suggest that lymphocyte may contribute to IL-16 up-regulation. However, the depletion of CD4^+^ cells, the most important population of lymphocytes detected by flow cytometry in CFA-inflamed paws, did not alter the increase in IL-16, thus indicating that this cell line is not a relevant source of IL-16. Probably, other types of cells could be involved in IL-16 increase during maintained inflammation. In this sense, the synthesis of IL-16 has been demonstrated in dendritic cells (Kaser et al. 1999) and, more related to inflammatory settings, it has been also described that fibroblasts may act as a source of IL-16 during rheumatoid arthritis (Franz et al. 1998). In any case, more data are necessary to determine which cells could cooperate with neutrophils to evoke the increase in IL-16 concentration observed in CFA-inflamed paws. Besides, apart from the outstanding role played by neutrophils in IL-16 production during inflammation, its abundant presence in inflamed tissues may suggest the possibility that these cells could also be involved in the response to IL-16 presence. The inhibition of IL-16-evoked hyperalgesia observed in mice in which neutrophils have been depleted following the administration of an anti-Ly6G antibody demonstrates that, effectively, neutrophils can be involved in IL-16 mediated hyperalgesia. These data, together with the inhibition of the hyperalgesia evoked by IL-16 when CD4 + cells are depleted may suggest that the population of neutrophils that express CD4 receptors (Biswas et al. 2003) could be crucial for this effect.

From a functional point of view, our results show that both thermal hyperalgesia and mechanical allodynia observed in acutely or chronically inflamed mice are dose-dependently counteracted by blocking the action of IL-16 with an anti-IL-16 antibody. Related to carrageenan-induced inflammation, thermal hyperalgesia assessed by the unilateral hot plate was completely inhibited at lower doses of the anti-IL-16 antibody than those necessary to evoke only a partial reduction of mechanical allodynia. In addition, the potency and efficacy of the anti-IL-16 antibody to revert the hypernociception derived from chronic inflammation was markedly greater. Thus, whereas the administration of 1 µg/kg of anti-IL-16 antibody was completely ineffective to inhibit carrageenan-induced hyperalgesia, the same dose completely prevented the hyperalgesia measured 4 days after CFA administration. This lack of effect of low doses of the antibody against carrageenan-induced hyperalgesia does not seem to be related to a particular property of the inflammation induced by this agent but to its acute nature. This idea is supported by the fact that the acute effect evoked some hours after CFA administration was not affected either by the administration of 1 µg/kg and this dose only became effective from 2 days after the injection of the inflammatory agent. The explanation for this difference is not easy, although it could be suggested that the lower concentration of IL-16 found in CFA-inflamed paws as compared to carrageenan-inflamed ones when performing ELISA assays in extracellular fluids could be an initial reason by which greater doses of antiÍL-16 antibody are necessary to neutralize its nociceptive effects.

The consideration that IL-16 could be a relevant molecule in inflammatory events as hyperalgesia and allodynia seems to fit well with our previous knowledge related to the molecular mechanisms involved in these settings. Initially, the involvement of PG synthesis and TRP channels sensitization in the hyperalgesic action of IL-16 is coherent with their notable role in inflammation and hyperalgesia. More data are necessary to precisely delineate the possible upstream mechanisms that could lead to the release of IL-16 from neutrophils during inflammation, although several molecules involved in inflammation could fulfil such a role. For instance, it has been described that mediators such as S1008, expressed after the administration of carrageenan (Goto et al. 2021) or CFA (Li et al. 2021), can favour the synthesis and release of IL-16 from neutrophils (Katano et al. 2011).

Altogether, our results indicate that IL-16 present in inflamed tissues functionally contributes to the instauration of hyperalgesia and allodynia and that this interleukin could be relevant in short-term inflammations with only several hours of evolution, as demonstrated by data obtained with the carrageenan model, or in more chronic situations, as that evoked by CFA. Accordingly, the present data also give support to the possibility that the neutralization of IL-16 with an antibody could be a useful strategy for alleviating inflammatory pain. In particular, the greater potency of the anti-IL-16 antibody to reduce nociceptive symptoms in the more long-lasting inflammation evoked by CFA could suggest that the blockade of IL-16 might be especially useful to counteract maintained inflammatory hypernociception. Reinforcing this view, just when this paper was being prepared in its final form, another report focused on the role of spinal IL-16 in CFA-induced hypernociception proposed that its antagonism may be a promising target for the treatment of inflammatory pain (Zhu et al. 2024). This publication demonstrates the increased presence of IL-16 in glial spinal cells in response to inflammation and, although peripheral modulation of nociception is not its central topic, it also describes that i.pl. administration of IL-16 can evoke mechanical allodynia measured by von Frey filaments. However, contrasting with our results, IL-16-induced thermal hyperalgesia was not observed after its i.pl. injection. Several methodological variables could account for this discrepancy. Thus, the results of the mentioned publication (Zhu et al. 2024) were obtained by assaying the effects of higher doses of IL-16 at longer times after injection than those used here. Moreover, the thermal test used was based in radiant heat whereas we use the unilateral hot plate test, in which the whole plantar surface is in contact with a heated surface and, as previously described, this fact can justify the obtaining of different responses (Menéndez et al. 2002). In any case, our results together with those of the cited manuscript contribute to establish a solid experimental framework supporting a key role for IL-16 in clinical inflammatory diseases, as could be rheumatoid arthritis (Cho et al. 2008; ElAtta et al. 2019). Looking forward, it seems advisable to perform more studies to elucidate whether IL-16 neutralization may be a pharmacological strategy specific for inflammation-related pathologies or might be also useful in different types of painful settings, as those derived from neuropathic or neoplastic injuries.

## Data Availability

Data will be provided upon request.
